# Caspase-3 Deletion Promotes Necrosis in Atherosclerotic Plaques of ApoE Knockout Mice

**DOI:** 10.1155/2016/3087469

**Published:** 2016-10-26

**Authors:** Mandy O. J. Grootaert, Dorien M. Schrijvers, Marthe Hermans, Viviane O. Van Hoof, Guido R. Y. De Meyer, Wim Martinet

**Affiliations:** ^1^Laboratory of Physiopharmacology, University of Antwerp, 2610 Antwerp, Belgium; ^2^Department of Clinical Chemistry, Antwerp University Hospital, 2610 Antwerp, Belgium

## Abstract

Apoptosis of macrophages and vascular smooth muscle cells (VSMCs) in advanced atherosclerotic plaques contributes to plaque progression and instability. Caspase-3, a key executioner protease in the apoptotic pathway, has been identified in human and mouse atherosclerotic plaques but its role in atherogenesis is not fully explored. We therefore investigated the impact of caspase-3 deletion on atherosclerosis by crossbreeding caspase-3 knockout (Casp3^−/−^) mice with apolipoprotein E knockout (ApoE^−/−^) mice. Bone marrow-derived macrophages and VSMCs isolated from Casp3^−/−^ApoE^−/−^ mice were resistant to apoptosis but showed increased susceptibility to necrosis. However, caspase-3 deficiency did not sensitize cells to undergo RIP1-dependent necroptosis. To study the effect on atherosclerotic plaque development, Casp3^+/+^ApoE^−/−^ and Casp3^−/−^ApoE^−/−^ mice were fed a western-type diet for 16 weeks. Though total plasma cholesterol, triglycerides, and LDL cholesterol levels were not altered, both the plaque size and percentage necrosis were significantly increased in the aortic root of Casp3^−/−^ApoE^−/−^ mice as compared to Casp3^+/+^ApoE^−/−^ mice. Macrophage content was significantly decreased in plaques of Casp3^−/−^ApoE^−/−^ mice as compared to controls, while collagen content and VSMC content were not changed. To conclude, deletion of caspase-3 promotes plaque growth and plaque necrosis in ApoE^−/−^ mice, indicating that this antiapoptotic strategy is unfavorable to improve atherosclerotic plaque stability.

## 1. Introduction

Apoptosis is a programmed form of cell death that occurs in all major cell types of atherosclerotic plaques where it is implicated in the development and progression of the disease [1–3]. Indeed, increasing evidence shows that vulnerable, rupture-prone plaques display higher levels of apoptosis than stable lesions [4, 5]. The apoptotic process is governed by cysteine aspartate specific proteases, termed caspases, that progressively disassemble the dying cell by the selective cleavage of cellular substrates involved in cytoskeleton reorganization and nuclear degradation [6]. In this way, apoptosis is characterized by cellular shrinkage, chromatin condensation, membrane blebbing, and finally internucleosomal DNA fragmentation [7]. Apoptosis may be induced by several proinflammatory (e.g., IFN*γ* and TNF*α*), prooxidative (ROS), and cytotoxic (hypoxia and cholesterol overload) stimuli present in atherosclerotic plaques [3, 8, 9]. Although cellular loss via apoptotic cell death has a fundamental impact on plaque stability, this effect depends on the stage of plaque development and the cell type that is involved. Apoptosis of vascular smooth muscle cells (VSMCs) promotes plaque vulnerability in both early and advanced lesions by disintegration of the protective fibrous cap [10], while macrophage apoptosis limits plaque growth in early lesions by hampering inflammation [11–13]. In advanced lesions, however, it promotes necrotic core formation and plaque instability [12, 13]. Because the phagocytic clearance of apoptotic cells is impaired in advanced plaques, apoptotic bodies accumulate and undergo secondary necrosis [14, 15]. An increased death of professional phagocytes such as macrophages further compromises apoptotic cell removal and promotes plaque growth [16].

Given the potential consequences of macrophage and VSMC apoptosis in advanced atherosclerosis, the development of different strategies to target apoptosis in atherosclerosis has been an important objective for many years. In the past decade, researchers have made considerable efforts to improve features of plaque stability in experimental mouse models by inhibiting apoptosis through genetic disruption of the death receptors or their ligands [17–20], the proapoptotic proteins of the mitochondrial pathway [21], or the tumor suppressor proteins [22–27]. However, up till now, caspases have never been explored as a suitable target to modulate apoptotic cell death in atherosclerosis. Because previous studies have identified cleaved caspase-3 in both human [28] and mouse [29] atherosclerotic plaques, where it colocalizes with dead macrophages and lipid-rich plaque components [30], we aimed to investigate the impact of caspase-3 deletion on atherogenesis by crossbreeding caspase-3 knockout (Casp3^−/−^) mice with ApoE knockout (ApoE^−/−^) mice. After 16 weeks on a western-type diet, Casp3^−/−^ApoE^−/−^ mice developed larger plaques that were characterized by increased plaque necrosis. The present study indicates that deletion of caspase-3 as a model for apoptosis deficiency promotes a switch to necrosis and may therefore not be a favorable strategy to improve atherosclerotic plaque stability.

## 2. Materials and Methods

### 2.1. Mice

Caspase-3 deficient (Casp3^−/−^) mice (C57BL/6; Jackson Laboratory; stock number 006233) were crossbred with atherosclerosis-prone apolipoprotein E deficient (ApoE^−/−^) mice (C57BL/6; Jackson Laboratory; stock number 002052). Male Casp3^+/+^ApoE^−/−^ (*n* = 8) and Casp3^−/−^ApoE^−/−^ (*n* = 7) mice were fed a western-type diet (WD) (4021.90, AB Diets) for 16 weeks to induce plaque formation. Mice were housed in a temperature-controlled room with a 12 h light/dark cycle and food and water ad libitum. After 16 weeks on WD, mice were anesthetized with sevoflurane (8% for induction and 4,5% for maintenance; SevoFlo®; Penlon vaporizer) to perform transthoracic echocardiograms using a Toshiba diagnostic ultrasound system (SSA-700A) equipped with a 15 MHz transducer. End-diastolic diameter (EDD) and end-systolic diameter (ESD) were measured and fractional shortening (FS) was calculated as follows: ([EDD − ESD]/EDD)*∗*100. Next, mice were fasted overnight and blood was collected by cardiac puncture under terminal anesthesia (Nembutal 250 mg/kg). Total cholesterol, LDL cholesterol, and triglyceride levels were measured on a Dimension Vista® System (Siemens Healthcare Diagnostics) with reagents from the same manufacturer. Tissues were fixed in formalin 4% for 24 h before paraffin imbedding. All experiments were approved by the Ethical Committee of the University of Antwerp.

### 2.2. Cell Culture

Bone marrow-derived macrophages (BMDM) were harvested by flushing bone marrow from the hind limbs of Casp3^+/+^ApoE^−/−^ and Casp3^−/−^ApoE^−/−^ mice. Cells were cultured for 7 d in RPMI medium (Gibco Life Technologies) supplemented with 15% L-cell conditioned medium (LCCM) containing monocyte-colony stimulating factor (M-CSF). VSMCs were isolated from the aorta as previously described [31–33]. Briefly, aortas were incubated in 1 mg/mL collagenase type II CLS (Worthington), 1 mg/mL soybean trypsin inhibitor (Worthington), and 0.74 units/mL elastase (Worthington) for 15 min at 37°C to remove the adventitia. Thereafter, the aortas were placed in fresh enzyme solution for 1 h. Isolated cells were collected, washed, and resuspended in DMEM/F-12 medium (Gibco Life Technologies) supplemented with 20% FBS (Sigma-Aldrich). To induce apoptosis, BMDM were treated with cycloheximide (Sigma-Aldrich) or oxLDL (medium oxidized LDL; Kalen Biomedical; 770202) for 8 h while VSMCs were treated with puromycin (Sigma-Aldrich) or oxLDL for 16 h. zVAD-fmk (Enzo Life Sciences) was used as a pan-caspase inhibitor. Apoptosis was monitored by a TUNEL staining (S7101, Millipore) and a caspase-3/7 activity fluorometric assay (K105-100; BioVision). Necrosis was monitored by propidium iodide (PI, Molecular Probes) labeling. Briefly, cells cultured in 12-well plates were labeled with Hoechst and PI at the end of the experiment and immediately visualized with fluorescence microscopy (EVOS® FL Cell Imaging System; ThermoFisher Scientific). In this way, cells are not manipulated and membrane integrity is preserved. PI+ and PI– cells were counted on 2 representative images per well (approx. 150 cells/image). The average value was calculated and used in the statistical analysis. Moreover, each condition was performed in duplicate. PI labeling experiments were conducted by two independent users. Necrostatin-1 (Nec-1; Enzo Life Sciences), a RIPK1 inhibitor, was used to inhibit necroptosis while the combination of 100 ng/mL LPS (Sigma-Aldrich) and 20 *μ*mol/L zVAD-fmk was used as a positive control for necroptosis induction.

### 2.3. Western Blotting

Cells were lysed in Laemmli sample buffer (Bio-Rad) containing *β*-mercaptoethanol (Sigma-Aldrich) and boiled for 4 min. Samples were loaded on Bolt 4–12% Bis-Tris gels (Life Technologies) and after electrophoresis transferred to Immobilon-FL membranes (Millipore). Membranes were probed with rabbit anti-cleaved caspase-3 (9661; Cell Signaling Technologies) or mouse anti-*β*-actin (A5441, clone AC-15; Sigma-Aldrich) primary antibodies. Subsequently, membranes were incubated with infrared- (IR-) conjugated secondary antibodies (IgG926-32211 (anti-rabbit); IgG926-68070 (anti-mouse); LI-COR Biosciences) to allow IR fluorescence detection using an Odyssey SA infrared imaging system (LI-COR Biosciences).

### 2.4. Histological Analysis

Atherosclerotic plaques located in the aortic root were analyzed in 4 different sections sliced at equally spaced intervals (every 50 *μ*m). Necrosis was measured on a hematoxylin-eosin (H&E) staining according to a standard method [34]. The percentage of necrosis was calculated as the size of the necrotic core divided by the total plaque size. A 3000 *μ*m^2^ minimum threshold was implemented in order to avoid counting of regions that likely do not represent substantial areas of necrosis. Total collagen was detected by a Sirius red staining (Sigma-Aldrich). Plaques were further analyzed by immunohistochemistry with rabbit anti-Mac-3 (553322; BD Pharmingen) and mouse anti-*α*-SMC-actin (A2547; Sigma-Aldrich) primary antibodies. Thereafter, tissue sections were incubated with species-appropriate HRP-conjugated secondary antibodies followed by 60 min of reactive ABC. 3,3′-Diaminobenzidine or 3-amino-9-ethyl-carbazole was used as a chromogen. All images were acquired with Universal Grab 6.1 software using an Olympus BX40 microscope and quantified with Image J software.

### 2.5. Statistical Analysis

All data were analyzed with SPSS 23.0 software (SPSS Inc.) and presented as mean ± SEM. Statistical tests are mentioned in the figure legends. Differences were considered significant at *P* < 0.05.

## 3. Results

### 3.1. Apoptosis is Inhibited in Caspase-3 Deficient Macrophages and Vascular Smooth Muscle Cells

**Figure 1:**
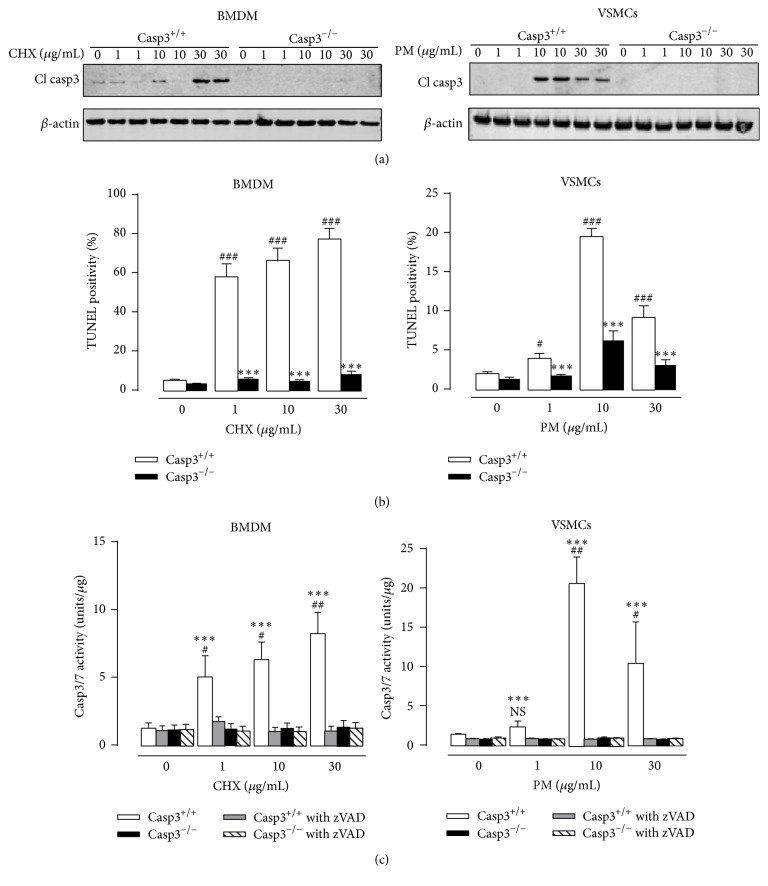
Apoptosis is inhibited in caspase-3 deficient macrophages and vascular smooth muscle cells. BMDM and VSMCs were isolated from Casp3^+/+^ApoE^−/−^ (Casp3^+/+^) and Casp3^−/−^ApoE^−/−^ (Casp3^−/−^) mice and treated with cycloheximide (CHX) (0–30 *μ*g/mL) and puromycin (PM) (0–30 *μ*g/mL), respectively. Apoptosis was monitored by (a) western blot analysis for cleaved caspase-3 (*β*-actin was used as loading control), (b) TUNEL staining (*n* = 3 independent experiments with 2 counting regions of 200 cells/region in duplicate; ^###^ *P* < 0.001 and ^#^ *P* < 0.05 versus 0 *μ*g/mL; ^*∗∗∗*^ *P* < 0.001 versus Casp3^+/+^; factorial ANOVA with genotype and treatment as category factors; Dunnett post hoc) and (c) caspase-3/7 fluorometric activity assay (*n* = 3 independent experiments; ^##^ *P* < 0.01, ^#^ *P* < 0.05, and NS, not significant, versus 0 *μ*g/mL; ^*∗∗∗*^ *P* < 0.001 versus Casp3^−/−^; factorial ANOVA with genotype and treatment as category factors; Dunnett post hoc).

Bone marrow-derived macrophages (BMDM) and aortic vascular smooth muscle cells (VSMCs) were isolated from Casp3^−/−^ApoE^−/−^ mice and treated with apoptosis inducers cycloheximide (CHX) (1 *μ*g/mL; 10 *μ*g/mL; 30 *μ*g/mL) and puromycin (PM) (1 *μ*g/mL; 10 *μ*g/mL; 30 *μ*g/mL), respectively, to validate their inability to undergo apoptosis. Western blot analysis confirmed the absence of cleaved caspase-3 in Casp3^−/−^ApoE^−/−^ BMDM and Casp3^−/−^ApoE^−/−^ VSMCs in both untreated and treated conditions (Figure 1(a)). Moreover, TUNEL staining showed a clear inhibition of DNA fragmentation in CHX-treated Casp3^−/−^ApoE^−/−^ BMDM and PM-treated Casp3^−/−^ApoE^−/−^ VSMCs as compared to Casp3^+/+^ApoE^−/−^ BMDM and Casp3^+/+^ApoE^−/−^ VSMCs (Figure 1(b)). To rule out the possibility that caspase-7, which shares similar substrate specificity with caspase-3, is compensatory activated in Casp3^−/−^ApoE^−/−^ BMDM and Casp3^−/−^ApoE^−/−^ VSMCs, a fluorometric DEVD substrate-based caspase activity assay was performed. In contrast to Casp3^+/+^ApoE^−/−^ BMDM and Casp3^+/+^ApoE^−/−^ VSMCs, treatment of Casp3^−/−^ApoE^−/−^ BMDM and Casp3^−/−^ApoE^−/−^ VSMCs with apoptosis inducers did not result in increased activity of caspase-3/7 (Figure 1(c)). Cotreatment with the pan-caspase-inhibitor zVAD-fmk completely abolished caspase-3/7 activity in CHX-treated Casp3^+/+^ApoE^−/−^ BMDM and PM-treated Casp3^+/+^ApoE^−/−^ VSMCs. However, zVAD-fmk did not further decrease caspase activity in CHX-treated Casp3^−/−^ApoE^−/−^ BMDM and PM-treated Casp3^−/−^ApoE^−/−^ VSMCs, indicating that there is no residual caspase-7 activity in caspase-3 deficient BMDM and VSMCs.

### 3.2. Caspase-3 Deficiency Triggers a Switch from Apoptosis to Necrosis

**Figure 2:**
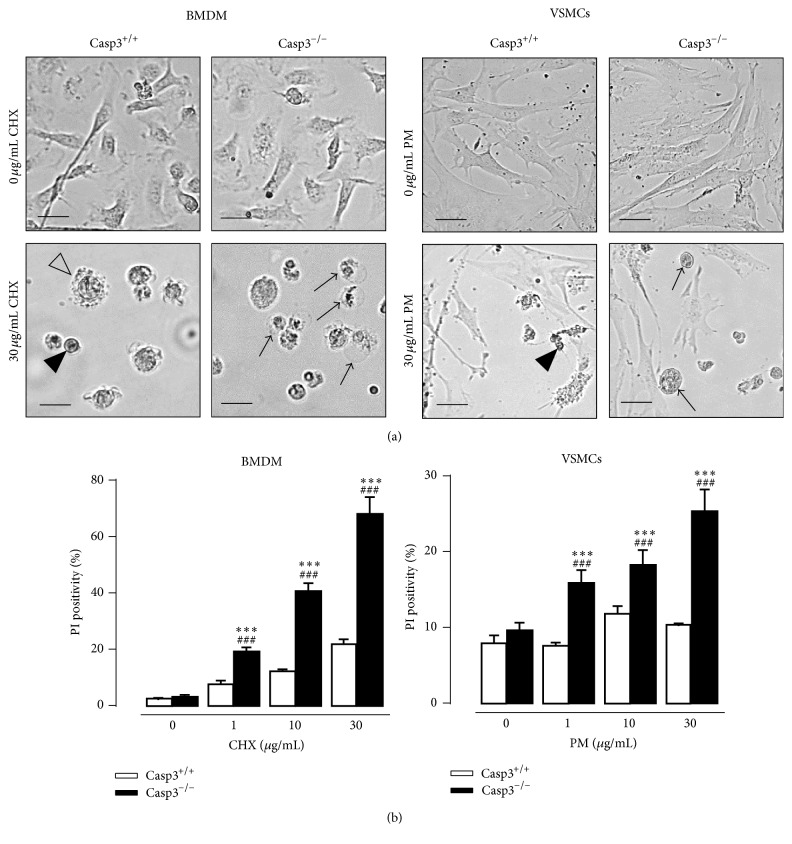
Caspase-3 deficiency in macrophages and vascular smooth muscle cells triggers a switch to necrosis in response to apoptotic stimuli. BMDM and VSMCs were isolated from Casp3^+/+^ApoE^−/−^ (Casp3^+/+^) and Casp3^−/−^ApoE^−/−^ (Casp3^−/−^) mice and treated with cycloheximide (CHX) (0–30 *μ*g/mL) and puromycin (PM) (0–30 *μ*g/mL), respectively. Necrosis was monitored by (a) morphological analysis (necrotic cells were characterized by cellular oncosis (black arrows) while apoptotic cells showed cellular shrinkage (black arrowheads) and membrane blebbing (open arrowhead)) (scale bar: 25 *μ*m (BMDM) and 50 *μ*m (VSMCs)) and (b) PI labeling (*n* = 3 independent experiments with 2 counting regions of 150 cells/region in duplicate; ^###^ *P* < 0.001 versus 0 *μ*g/mL; ^*∗∗∗*^ *P* < 0.001 versus Casp3^+/+^; factorial ANOVA with genotype and treatment as category factors; Dunnett post hoc).

Casp3^−/−^ApoE^−/−^ BMDM treated with the apoptosis inducer CHX were not protected against cell death but underwent necrosis (Figure 2(a) left panel). According to a morphological analysis, CHX-treated Casp3^−/−^ApoE^−/−^ BMDM were characterized by cell oncosis (i.e., increased cell volume) and a translucent cytoplasm, indicative of necrosis. In contrast, Casp3^+/+^ApoE^−/−^ BMDM showed membrane blebbing and cellular shrinkage upon CHX treatment, which are typical characteristics of apoptotic cell death. Moreover, CHX-treated Casp3^−/−^ApoE^−/−^ BMDM were characterized by a loss of membrane integrity as shown by increased PI labeling (Figure 2(b) left panel). Consistent with BMDM, Casp3^−/−^ApoE^−/−^ VSMCs showed increased susceptibility to necrosis when treated with the apoptosis inducer PM as illustrated by increased cell oncosis (Figure 2(a) right panel) and PI labeling (Figure 2(b) right panel).

**Figure 3:**
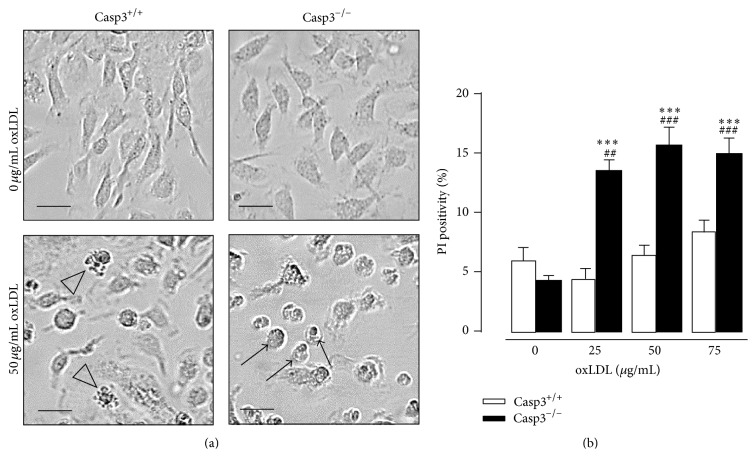
Caspase-3 deficiency in macrophages triggers a switch to necrosis in response to the atherosclerosis-related stimulus oxLDL. BMDM were isolated from Casp3^+/+^ApoE^−/−^ (Casp3^+/+^) and Casp3^−/−^ApoE^−/−^ (Casp3^−/−^) mice and treated with oxLDL (25–75 *μ*g/mL). Necrosis was monitored by (a) morphological analysis (necrotic cells were characterized by cellular oncosis (black arrows) while apoptotic cells showed membrane blebbing (open arrowhead)) (scale bar: 25 *μ*m (BMDM)) and (b) PI labeling (*n* = 3 independent experiments with 2 counting regions of 150 cells/region in duplicate; ^###^ *P* < 0.001 and ^##^ *P* < 0.01 versus 0 *μ*g/mL; ^*∗∗∗*^ *P* < 0.001 versus Casp3^+/+^; factorial ANOVA with genotype and treatment as category factors; Dunnett post hoc).

To explore whether a similar switch to necrosis occurs in response to an atherosclerosis-related stimulus, cells were treated with medium oxidized LDL (oxLDL). Casp3^−/−^ApoE^−/−^ BMDM showed increased susceptibility to necrosis when treated with oxLDL while Casp3^+/+^ApoE^−/−^ BMDM displayed clear signs of apoptosis (Figures 3(a) and 3(b)). However, oxLDL did not trigger apoptosis in Casp3^+/+^ApoE^−/−^ VSMCs based on the absence of typical apoptotic morphological changes and lack of cleavage of caspase-3 and caspase-7 (data not shown). In this way, the possible switch to necrosis in oxLDL-treated Casp3^−/−^ApoE^−/−^ VSMCs could not be determined.

**Figure 4:**
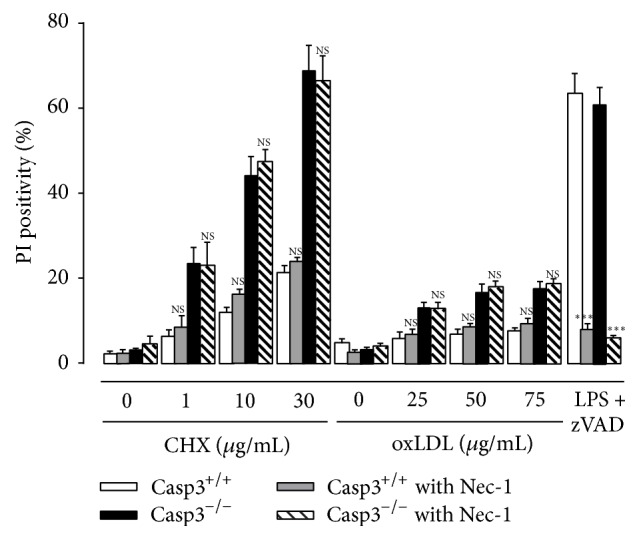
Caspase-3 deficiency in macrophages does not trigger a switch to necroptosis. BMDM were isolated from Casp3^+/+^ApoE^−/−^ (Casp3^+/+^) and Casp3^−/−^ApoE^−/−^ (Casp3^−/−^) mice and treated with cycloheximide (CHX) (0–30 *μ*g/mL) or oxLDL (25–75 *μ*g/mL) in the presence or absence of the necroptosis inhibitor Necrostatin-1 (Nec-1) (30 *μ*mol/L). BMDM treated with 100 ng/mL LPS and 20 *μ*mol/L zVAD-fmk were used as positive control for necroptosis induction. Necrosis was monitored by PI labeling (*n* = 3 independent experiments with 2 counting regions of 150 cells/region in duplicate; NS, not significant; ^*∗∗∗*^ *P* < 0.001 versus Casp3^+/+^ and Casp3^−/−^ without Nec-1; factorial ANOVA with genotype and treatment as category factors; Dunnett post hoc).

To investigate whether the observed necrosis in CHX- and oxLDL-treated Casp3^−/−^ApoE^−/−^ BMDM can be classified as necroptosis (i.e., programmed necrosis), PI labeling experiments were conducted in the presence or absence of Necrostatin-1 (Nec-1), a well-known necroptosis inhibitor (Figure 4). Cotreatment with Nec-1 did not affect the degree of necrosis in Casp3^−/−^ApoE^−/−^ BMDM, indicating that caspase-3 deficiency does not trigger a switch to necroptosis. Of note, treatment of Casp3^−/−^ApoE^−/−^ and Casp3^+/+^ApoE^−/−^ BMDM with the necroptosis inducer LPS/zVAD-fmk showed that Casp3^−/−^ApoE^−/−^ BMDM were as efficient as Casp3^+/+^ApoE^−/−^ BMDM to undergo necroptosis.

### 3.3. Caspase-3 Deficiency Increases Total Plaque Size and Plaque Necrosis in ApoE^−/−^ Mice

To investigate the role of caspase-3 on atherosclerosis, Casp3^+/+^ApoE^−/−^ and Casp3^−/−^ApoE^−/−^ mice were fed a western-type diet for 16 weeks. Body weight, heart weight, total plasma cholesterol, LDL cholesterol, and triglycerides were not different between Casp3^+/+^ApoE^−/−^ mice and Casp3^−/−^ApoE^−/−^ mice (Table 1). Echocardiography did not reveal any signs of left ventricle dilatation or impaired cardiac function as a result of caspase-3 deficiency (Table 1).

**Figure 5:**
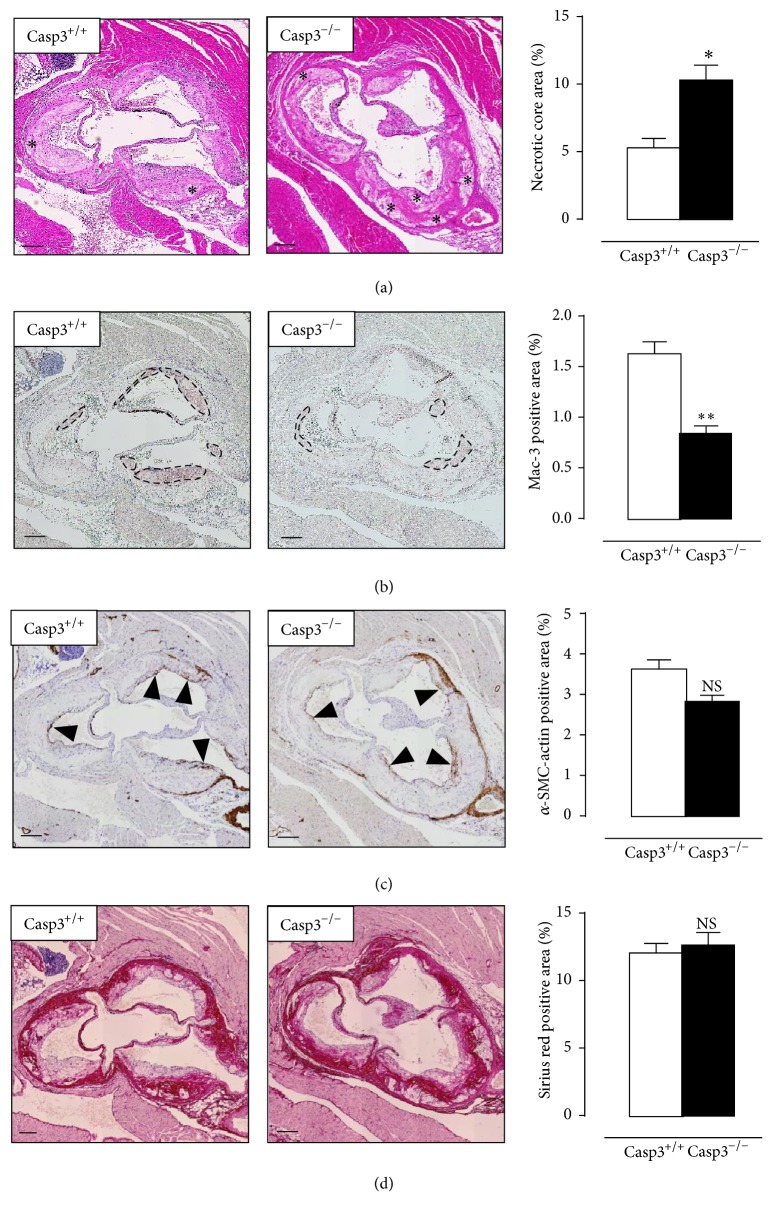
Caspase-3 deficiency increases plaque necrosis in ApoE^−/−^ mice. Casp3^+/+^ApoE^−/−^ mice (Casp3^+/+^) (*n* = 8) and Casp3^−/−^ApoE^−/−^ mice (Casp3^−/−^) (*n* = 7) were fed a western-type diet for 16 weeks. (a) Aortic root atherosclerotic plaques were stained with H&E to quantify plaque necrosis (the necrotic cores are indicated by an asterisk). (b, c) Serial sections were immunostained for Mac-3 and *α*-SMC-actin to determine macrophage content (Mac-3 positive regions are indicated by black circles) and vascular smooth muscle cell content (*α*-SMC-actin positive fibrous caps are indicated by black arrowheads), respectively. (d) Serial sections were stained with Sirius red to quantify total collagen content (characterized by intense pink/red staining). (^*∗*^ *P* < 0.05, ^*∗∗*^ *P* < 0.01, and NS, not significant, versus Casp3^+/+^; repeated measures) scale bar: 150 *μ*m.

Atherosclerotic plaques located in the aortic root of Casp3^−/−^ApoE^−/−^ mice (144 ± 8 × 10^3^ *μ*m^2^) were significantly larger as compared to Casp3^+/+^ApoE^−/−^ mice (108 ± 6 × 10^3^ *μ*m^2^) and were characterized by increased plaque necrosis (Figure 5(a)). The necrotic cores in plaques of Casp3^−/−^ApoE^−/−^ mice were not only larger but also more abundantly present. Furthermore, plaque macrophage content was significantly reduced in Casp3^−/−^ApoE^−/−^ mice (Figure 5(b)) while smooth muscle cell content and collagen content were not different between both groups (Figures 5(c) and 5(d)).

## 4. Discussion

Given the detrimental consequences of macrophage and VSMC apoptosis in advanced atherosclerosis, further improving our knowledge on the regulation of apoptosis in atherosclerosis is imperative to explore the potential of antiapoptotic plaque stabilizing strategies. Because caspases are an unexplored yet attractive target to modulate apoptotic cell death in atherosclerosis, we chose to examine the impact of caspase-3 deletion on atherosclerosis by crossbreeding caspase-3 knockout (Casp3^−/−^) mice with ApoE knockout (ApoE^−/−^) mice. Bone marrow-derived macrophages (BMDM) and vascular smooth muscle cells (VSMCs), two major constituents of atherosclerotic plaques, were isolated from Casp3^+/+^ApoE^−/−^ mice and Casp3^−/−^ApoE^−/−^ mice and subjected to the apoptosis inducers cycloheximide [35] or puromycin [36], respectively, to validate our experimental model. Caspase-3 deficient BMDM and VSMCs showed resistance to apoptosis as illustrated by reduced TUNEL positivity. Moreover, deletion of caspase-3 did not evoke a compensatory activation of caspase-7 in Casp3^−/−^ApoE^−/−^ macrophages and VSMCs, despite their shared substrate specificity. These experiments underline the critical role of caspase-3 in the execution of apoptotic cell death and verify the successful inhibition of apoptosis in this model. Nevertheless, caspase-3 deficient BMDM and VSMCs were not resistant to cell death but instead showed increased susceptibility to necrosis upon exposure to CHX/PM as detected by increased cell oncosis and PI labeling. The observed switch between apoptotic cell death and necrotic cell death in antiapoptotic conditions has been reported previously by others [37–39] and affirms that both cell death pathways share a common biochemical network. Similar results were obtained in Casp3^−/−^ApoE^−/−^ macrophages when treated with the atherosclerosis-related stimulus oxLDL. However, according to morphological analysis and western blot analysis of cleaved caspase-3 and cleaved caspase-7, oxLDL (25–75 *μ*g/mL) did not induce apoptosis in Casp3^+/+^ApoE^−/−^ VSMCs. Therefore, it was impossible to determine a potential switch to necrosis in Casp3^−/−^ApoE^−/−^ VSMCs. The observed resistance of Casp3^+/+^ApoE^−/−^ VSMCs to apoptosis induced by oxLDL can be explained by different factors including the degree of oxidation and the concentration of oxLDL. The latter can be excluded since increasing the concentration up to 150 *μ*g/mL did not further reduce cell viability (data not shown). Previous reports have shown that oxLDL may exert dual effects on VSMCs depending on its degree of oxidation [40, 41]. Moreover, differences in pathways of oxLDL uptake might affect the intracellular concentration of oxLDL. According to a recent report, VSMCs take up modified LDL via different mechanisms as compared to macrophages [42] and may consequently trigger alternative signaling cascades. Our experiments suggest that VSMCs are intrinsically less susceptible to the cytotoxic effects of medium oxidized LDL than macrophages. To investigate whether the observed necrosis in Casp3^−/−^ApoE^−/−^ BMDM can be classified as necroptosis (i.e., programmed necrosis regulated by RIP1 and RIP3), PI labeling experiments were conducted in the presence or absence of the necroptosis inhibitor Necrostatin-1 (Nec-1). According to literature, knockdown of caspase-8 in L929 cells triggers TNF*α*-induced necroptosis due to the absence of caspase-8-mediated inactivation of RIP1 and RIP3 [43]. In the present study, treatment of CHX- and oxLDL-treated macrophages with the necroptosis inhibitor Necrostatin-1 did not influence the degree of necrosis, indicating that caspase-3, in contrast to caspase-8, does not play a role in the regulation of necroptosis. Taken together, caspase-3 deletion protects macrophages and smooth muscle cells against apoptosis but triggers a switch to necrosis. These data indicate that caspase-3 plays a crucial role in the execution of apoptosis, though deletion of caspase-3 does not prevent cell death.

To study the consequences of caspase-3 deletion on plaque development, Casp3^+/+^ApoE^−/−^ and Casp3^−/−^ApoE^−/−^ mice were fed a western-type diet for 16 weeks. Plasma cholesterol, triglycerides, LDL cholesterol levels, and cardiac parameters (EDD, ESD, and FS) were not different between Casp3^−/−^ApoE^−/−^ mice and Casp3^+/+^ApoE^−/−^ mice, indicating that deletion of caspase-3 affected neither lipid metabolism nor cardiac function. Analysis of atherosclerotic plaques showed that Casp3^−/−^ApoE^−/−^ mice developed larger plaques that were characterized by increased plaque necrosis as compared to plaques of Casp3^+/+^ApoE^−/−^ mice. With regard to the cellular composition, plaques of Casp3^−/−^ApoE^−/−^ mice showed a significant decrease in macrophage content and a trend towards reduced VSMC content as compared to Casp3^+/+^ApoE^−/−^ plaques. The decrease in macrophage content most likely reflects the observed rise in plaque necrosis. Necrotic macrophages accumulate in the necrotic core while losing their cell-type specific epitopes, hindering their detection. These findings are in line with previous observations in mice lacking the proapoptotic protein Bax [21], the death receptors TNFR1 [17] and Fas [20], or the tumor suppressor proteins p53 [22–26] and p19(ARF) [27]. Macrophage specific deletion of Bax reduces macrophage apoptosis and stimulates the development of advanced atherosclerotic plaques in LDL receptor knockout mice [21]. Knockout of TNFR1 accelerates atherosclerosis in western-type diet-fed mice due to increased foam cell formation, though the effect on apoptosis and necrosis was not determined [17]. Impairment of Fas-mediated apoptosis, by targeting the Fas receptor [20] or its ligand [18], also accelerates atherosclerosis in ApoE^−/−^ mice. Genetic disruption of p53, either generally or macrophage-specifically, promotes atherosclerosis in ApoE^−/−^ mice [22, 25] and is often accompanied by increased plaque necrosis [23, 24, 26]. Moreover, deletion of the tumor suppressor p19(ARF) in ApoE^−/−^ mice reduces apoptosis in plaque macrophages and VSMCs and aggravates atherosclerosis, though the effect on plaque necrosis was not examined [27]. Based on our data and the reports from other groups, long-term inhibition of apoptosis might not be an attractive approach to fight chronic diseases such as atherosclerosis given the likelihood of promoting other, more harmful cell death routes. Also the potential risk of promoting tumorigenesis as a result of apoptosis inhibition must be considered. However, targeted, local delivery of the apoptosis inhibitor might limit this possibility [44]. Moreover, inhibition of caspase-3, in particular, might elicit undesirable effects and increase the risk for toxicity by interfering with its nonapoptotic physiological roles in cell differentiation [45]. Nevertheless, different animal studies have provided evidence that targeting apoptosis could be a valid strategy to treat heart failure (reviewed in [44]). Administration of broad-spectrum caspase inhibitors reduces myocardial infarct size in mice following ischemia reperfusion [46–48]. Conversely, cardiac overexpression of caspase-3 increases infarct size and aggravates cardiac dysfunction [49]. Hence, acute pharmacological inhibition of caspases, in particular caspase-3, still holds promise as a treatment for ischemia-related diseases where the cell death stimulus is only present for a short period of time and clearly differs from the long-term effects of caspase-3 deficiency.

## 5. Conclusion

Deletion of caspase-3 inhibits apoptosis in murine macrophages and vascular smooth muscle cells but triggers a switch to necrosis. Moreover, caspase-3 deficiency promotes plaque growth and plaque necrosis in 16-week western-type diet-fed ApoE^−/−^ mice. Considering the proinflammatory and prothrombotic properties of plaque necrosis [50], long-term inhibition of apoptosis may not be a favorable strategy to improve atherosclerotic plaque stability.

## Figures and Tables

**Table 1:** Characteristics of Casp3^+/+^ApoE^−/−^ mice and Casp3^−/−^ApoE^−/−^ mice after 16 weeks on western-type diet.

	Casp3^+/+^ApoE^−/−^	Casp3^−/−^ApoE^−/−^
*General*		
Body weight (g)^a^	34 ± 1	33 ± 1
Heart weight (mg)^a^	150 ± 3	161 ± 16
*Lipids*		
Total cholesterol (mg/dL)^a^	473 ± 32	423 ± 38
LDL cholesterol (mg/dL)^a^	309 ± 21	258 ± 29
Triglycerides (mg/dL)^a^	114 ± 7	105 ± 18
*Cardiac function *		
EDD LV (mm)^a^	4.2 ± 0.1	4.0 ± 0.2
ESD LV (mm)^a^	2.7 ± 0.1	2.6 ± 0.1
FS (%)^a^	36 ± 2	35 ± 1

^a^ Student's *t*-test; *P *> 0.05.

## References

[1] Kockx M. M. (1998). Apoptosis in the atherosclerotic plaque: quantitative and qualitative aspects. *Arteriosclerosis, Thrombosis, and Vascular Biology*.

[2] Mallat Z., Tedgui A. (2001). Current perspective on the role of apoptosis in atherothrombotic disease. *Circulation Research*.

[3] Stoneman V. E. A., Bennett M. R. (2004). Role of apoptosis in atherosclerosis and its therapeutic implications. *Clinical Science*.

[4] Kolodgie F. D., Narula J., Burke A. P. (2000). Localization of apoptotic macrophages at the site of plaque rupture in sudden coronary death. *The American Journal of Pathology*.

[5] Geng Y.-J., Libby P. (2002). Progression of atheroma: a struggle between death and procreation. *Arteriosclerosis, Thrombosis, and Vascular Biology*.

[6] Nicholson D. W., Thornberry N. A. (1997). Caspases: killer proteases. *Trends in Biochemical Sciences*.

[7] Kerr J. F., Wyllie A. H., Currie A. R. (1972). Apoptosis: a basic biological phenomenon with wide-ranging implications in tissue kinetics. *British Journal of Cancer*.

[8] Kockx M. M., Herman A. G. (2000). Apoptosis in atherosclerosis: beneficial or detrimental?. *Cardiovascular Research*.

[9] Mallat Z., Tedgui A. (2000). Apoptosis in the vasculature: mechanisms and functional importance. *British Journal of Pharmacology*.

[10] Clarke M. C. H., Figg N., Maguire J. J. (2006). Apoptosis of vascular smooth muscle cells induces features of plaque vulnerability in atherosclerosis. *Nature Medicine*.

[11] Arai S., Shelton J. M., Chen M. (2005). A role for the apoptosis inhibitory factor AIM/Sp*α*/Api6 in atherosclerosis development. *Cell Metabolism*.

[12] Stoneman V., Braganza D., Figg N. (2007). Monocyte/macrophage suppression in CD11b diphtheria toxin receptor transgenic mice differentially affects atherogenesis and established plaques. *Circulation Research*.

[13] Gautier E. L., Huby T., Witztum J. L. (2009). Macrophage apoptosis exerts divergent effects on atherogenesis as a function of lesion stage. *Circulation*.

[14] Schrijvers D. M., De Meyer G. R. Y., Herman A. G., Martinet W. (2007). Phagocytosis in atherosclerosis: molecular mechanisms and implications for plaque progression and stability. *Cardiovascular Research*.

[15] Seimon T., Tabas I. (2009). Mechanisms and consequences of macrophage apoptosis in atherosclerosis. *The Journal of Lipid Research*.

[16] Van Vré E. A., Ait-Oufella H., Tedgui A., Mallat Z. (2012). Apoptotic cell death and efferocytosis in atherosclerosis. *Arteriosclerosis, Thrombosis, and Vascular Biology*.

[17] Schreyer S. A., Peschon J. J., LeBoeuf R. C. (1996). Accelerated atherosclerosis in mice lacking tumor necrosis factor receptor p55. *The Journal of Biological Chemistry*.

[18] Aprahamian T., Rifkin I., Bonegio R. (2004). Impaired clearance of apoptotic cells promotes synergy between atherogenesis and autoimmune disease. *The Journal of Experimental Medicine*.

[19] Brånén L., Hovgaard L., Nitulescu M., Bengtsson E., Nilsson J., Jovinge S. (2004). Inhibition of tumor necrosis factor-*α* reduces atherosclerosis in apolipoprotein E knockout mice. *Arteriosclerosis, Thrombosis, and Vascular Biology*.

[20] Feng X., Li H., Rumbin A. A. (2007). ApoE^−/−^Fas^−/−^ C57BL/6 mice: a novel murine model simultaneously exhibits lupus nephritis, atherosclerosis, and osteopenia. *Journal of Lipid Research*.

[21] Liu J., Thewke D. P., Su Y. R., Linton M. F., Fazio S., Sinensky M. S. (2005). Reduced macrophage apoptosis is associated with accelerated atherosclerosis in low-density lipoprotein receptor-null mice. *Arteriosclerosis, Thrombosis, and Vascular Biology*.

[22] Guevara N. V., Kim H.-S., Antonova E. I., Chan L. (1999). The absence of p53 accelerates atherosclerosis by increasing cell proliferation in vivo. *Nature Medicine*.

[23] van Vlijmen B. J. M., Gerritsen G., Franken A. L. (2001). Macrophage p53 deficiency leads to enhanced atherosclerosis in APOE^∗^3-Leiden transgenic mice. *Circulation Research*.

[24] Merched A. J., Williams E., Chan L. (2003). Macrophage-specific p53 expression plays a crucial role in atherosclerosis development and plaque remodeling. *Arteriosclerosis, Thrombosis, and Vascular Biology*.

[25] Mercer J., Figg N., Stoneman V., Braganza D., Bennett M. R. (2005). Endogenous p53 protects vascular smooth muscle cells from apoptosis and reduces atherosclerosis in ApoE knockout mice. *Circulation Research*.

[26] Boesten L. S. M., Zadelaar A. S. M., van Nieuwkoop A. (2009). Macrophage p53 controls macrophage death in atherosclerotic lesions of apolipoprotein E deficient mice. *Atherosclerosis*.

[27] González-Navarro H., Abu Nabah Y. N., Vinué Á. (2010). p19ARF deficiency reduces macrophage and vascular smooth muscle cell apoptosis and aggravates atherosclerosis. *Journal of the American College of Cardiology*.

[28] Mallat Z., Ohan J., Lesèche G., Tedgui A. (1997). Colocalization of CPP-32 with apoptotic cells in human atherosclerotic plaques. *Circulation*.

[29] Nhan T. Q., Liles W. C., Chait A., Fallon J. T., Schwartz S. M. (2003). The p17 cleaved form of caspase-3 is present within viable macrophages in vitro and in atherosclerotic plaque. *Arteriosclerosis, Thrombosis, and Vascular Biology*.

[30] Hutter R., Valdiviezo C., Sauter B. V. (2004). Caspase-3 and tissue factor expression in lipid-rich plaque macrophages: evidence for apoptosis as link between inflammation and atherothrombosis. *Circulation*.

[31] Owens G. K., Loeb A., Gordon D., Thompson M. M. (1986). Expression of smooth muscle-specific *α*-isoactin in cultured vascular smooth muscle cells: relationship between growth and cytodifferentiation. *Journal of Cell Biology*.

[32] Geisterfer A. A. T., Peach Owens M. J. G. K. (1988). Angiotensin II induces hypertrophy, not hyperplasia, of cultured rat aortic smooth muscle cells. *Circulation Research*.

[33] Grootaert M. O., da Costa Martins P. A., Bitsch N. (2015). Defective autophagy in vascular smooth muscle cells accelerates senescence and promotes neointima formation and atherogenesis. *Autophagy*.

[34] Seimon T. A., Wang Y., Han S. (2009). Macrophage deficiency of p38*α* MAPK promotes apoptosis and plaque necrosis in advanced atherosclerotic lesions in mice. *Journal of Clinical Investigation*.

[35] Croons V., Martinet W., Herman A. G., Timmermans J.-P., De Meyer G. R. Y. (2007). Selective clearance of macrophages in atherosclerotic plaques by the protein synthesis inhibitor cycloheximide. *Journal of Pharmacology and Experimental Therapeutics*.

[36] Croons V., Martinet W., Herman A. G., De Meyer G. R. Y. (2008). Differential effect of the protein synthesis inhibitors puromycin and cycloheximide on vascular smooth muscle cell viability. *Journal of Pharmacology and Experimental Therapeutics*.

[37] Scheller C., Knöferle J., Ullrich A. (2006). Caspase inhibition in apoptotic T cells triggers necrotic cell death depending on the cell type and the proapoptotic stimulus. *Journal of Cellular Biochemistry*.

[38] Lemaire C., Andréau K., Souvannavong V., Adam A. (1998). Inhibition of caspase activity induces a switch from apoptosis to necrosis. *FEBS Letters*.

[39] Meilhac O., Escargueil-Blanc I., Thiers J.-C., Salvayre R., Nègre-Salvayre A. (1999). Bcl-2 alters the balance between apoptosis and necrosis, but does not prevent cell death induced by oxidized low density lipoproteins. *FASEB Journal*.

[40] Auge N., Pieraggi M.-T., Thiers J.-C., Negre-Salvayre A., Salvayre R. (1995). Proliferative and cytotoxic effects of mildly oxidized low-density lipoproteins on vascular smooth-muscle cells. *Biochemical Journal*.

[41] Björkerud B., Björkerud S. (1996). Contrary effects of lightly and strongly oxidized LDL with potent promotion of growth versus apoptosis on arterial smooth muscle cells, macrophages, and fibroblasts. *Arteriosclerosis, Thrombosis, and Vascular Biology*.

[42] Dubland J. A., Francis G. A. (2016). So Much Cholesterol: the unrecognized importance of smooth muscle cells in atherosclerotic foam cell formation. *Current Opinion in Lipidology*.

[43] Vanlangenakker N., Bertrand M. J. M., Bogaert P., Vandenabeele P., Vanden Berghe T. (2011). TNF-induced necroptosis in L929 cells is tightly regulated by multiple TNFR1 complex i and II members. *Cell Death and Disease*.

[44] Yang B., Ye D., Wang Y. (2013). Caspase-3 as a therapeutic target for heart failure. *Expert Opinion on Therapeutic Targets*.

[45] Fischer U., Schulze-Osthoff K. (2005). Apoptosis-based therapies and drug targets. *Cell Death and Differentiation*.

[46] Yaoita H., Ogawa K., Maehara K., Maruyama Y. (1998). Attenuation of ischemia/reperfusion injury in rats by a caspase inhibitor. *Circulation*.

[47] Holly T. A., Drincic A., Byun Y. (1999). Caspase inhibition reduces myocyte cell death induced by myocardial ischemia and reperfusion in vivo. *Journal of Molecular and Cellular Cardiology*.

[48] Huang J.-Q., Radinovic S., Rezaiefar P., Black S. C. (2000). In vivo myocardial infarct size reduction by a caspase inhibitor administered after the onset of ischemia. *European Journal of Pharmacology*.

[49] Condorelli G., Roncarati R., Ross J. (2001). Heart-targeted overexpression of caspase3 in mice increases infarct size and depresses cardiac function. *Proceedings of the National Academy of Sciences of the United States of America*.

[50] Martinet W., Schrijvers D. M., De Meyer G. R. Y. (2011). Necrotic cell death in atherosclerosis. *Basic Research in Cardiology*.

